# Advanced Nanomechanical Characterization of Biopolymer Films Containing GNPs and MWCNTs in Hybrid Composite Structure

**DOI:** 10.3390/nano12040709

**Published:** 2022-02-21

**Authors:** Todor Batakliev, Evgeni Ivanov, Verislav Angelov, Giovanni Spinelli, Rumiana Kotsilkova

**Affiliations:** 1 Open Laboratory on Experimental Micro and Nano Mechanics (OLEM), Institute of Mechanics, Bulgarian Academy of Sciences, Acad. G. Bonchev Str., Block 4, 1113 Sofia, Bulgaria; ivanov_evgeni@yahoo.com (E.I.); verislav@abv.bg (V.A.); spinelligiovanni76@gmail.com (G.S.); kotsilkova@yahoo.com (R.K.); 2 Research and Development of Nanomaterials and Nanotechnologies (NanoTech Lab Ltd.), Acad. G. Bonchev Str., Block 4, 1113 Sofia, Bulgaria; 3 Faculty of Transport Sciences and Technologies, University of Study “Giustino Fortunato”, Via Raffaele Delcogliano 12, 82100 Benevento, Italy

**Keywords:** biopolymer, carbon nanofillers, quasi-static nanoindentation, property mapping, nanoscratch, nanoDMA

## Abstract

Nanomechanical definition of the properties of composite specimens based on polylactic acid (PLA) was made in the present study. Research activities with accent on biodegradable polymer nanocomposites have fundamental significance originated from the worldwide plastic waste pollution. To receive hybrid nanocomposites with high level of homogeneity, the low cost and environmentally friendly melt extrusion method has been applied. The role of graphene nanoplatelets (GNPs) and multiwall carbon nanotubes (MWCNTs) as reinforcing nanoparticles dispersed in the polymer matrix was thoroughly investigated. Quasi-static nanoindentation analysis was enriched by performance of accelerated property mapping and nanodynamic mechanical testing in order to fully describe the nanoscale surface homogeneity and stress relaxation behavior of the nanocomposite specimens. That novelty of the research approach had a well-marked contribution over the detection of the new samples’ nanomechanical features as a function of the type of carbon nanofiller. Refined nanoscratch experiments uncovered the resistance of the materials against notches by means of measurement of the coefficient of friction and accurate estimation of the residual penetration depth.

## 1. Introduction

Nowadays, the nanomechanical test methods present one of the main solution for surface characterization of polymeric films and coatings [1,2,3,4]. Use of biodegradable polymer matrix as polylactic acid [5] has to be encouraged because it is a fair green alternative of the enormous quantity of plastic waste that is thrown out every day all over the earth. Numerous advantages of melt extrusion manufacturing method of nanocomposite materials with respect to attainment of better carbon nanoparticles dispersion have been already highlighted [6]. Composite specimens, prepared by melt blending, are investigated in order to see the effect of reinforcing nanoparticles on the crystallization kinetics of PLA die which is linked to the mechanical behavior and degradation rate of the samples [7]. The quality and the reliability of these advanced materials including the strengthening effect of GNPs and MWCNTs mixed in a polymer matrix can be ascertained only by blending of several advanced experimental approaches based on nanoscale indentation and scratch. Dynamic and static nanomechanical properties investigation of chemically modified clay-polymer nanocomposite revealed positive effect of both organic agents and clay loading on key parameters as elastic modulus, storage modulus, loss modulus, and loss factor [8]. Nanodynamic mechanical analysis (nanoDMA) has been used in the investigation of the physical features of carbon nanotubes (CNT)-filled composite material [9], in thermal stability researching of PLA/graphene nanocomposites [10], and yet in study of the viscoelastic behavior of natural fiber cell wall layers affected by nanoscale structural inhomogeneities in polymer composites [11]. Serious attention must be steered to the damping conduct of reinforced materials having in mind their wide industrial application in dynamic systems [12]. The authors of that article reported highest enhancement of tan δ values upon addition of 2 wt% CNTs in copolymer of L-lactide and ε-caprolactone. This damping behavior is explained with some conformational and morphological particularities of carbon and boron nitride nanotubes in accordance to an interfacial shear strength model [13]. Nanoindentation elastic modulus of copolymer composite matrix and the pullout as energy dissipation mechanism in composite materials containing nanotubes were found to be dependent on the level of uniform dispersion of CNTs within the polymer die [12,14]. Quantitative nanoindentation in combination with other physical methods demonstrated promising results in the study of the mechanics of soft materials at nanoscale by measuring storage and loss modulus as a function of oscillation frequency and time [15]. Stress relaxation behavior of biodegradable polymer films, modified via nanoparticle reinforcement, has been analyzed by conducting dynamic nanomechanical experiments at frequency sweep mode [16].

The current work is focused on seminal nanomechanical studies to provide the inputs for affirming our approach via five nanocomposite materials, each one holding GNPs and/or MWCNTs, that both render specific impact on the mechanical performance of the specimens. Our investigation was induced by the lack in the thematic literature of such complex nanomechanical research on PLA-based nanocomposite samples. The purpose of the work is to expand the knowledge related to the above mentioned reinforcement of a biopolymer matrix by incorporation of carbon nanofillers and creation of hybrid composite structure.

## 2. Materials and Methods

### 2.1. Materials

The basic biopolymer—Ingeo™ PLA grade 3D850 having melt flow rate of 7–9 g/10 min (210 °C, 2.16 kg) was purchased from Nature Works (Minnetonka, MN, USA). Two commercial carbon nanofillers covering the requirement for optimal ratio of price vs. quality were selected to be used in the process of melt blending and subsequent preparation of two PLA-based masterbatches containing 6 wt%GNP and 6 wt%MWCNT. These monofiller composites served for receiving of three bifiller compositions with carbon filler wt% ratios of 3:3; 1.5:4.5, and 4.5:1.5 GNPs to MWCNTs. GNPs (Industrial Graphene Nanoplatelets, TNIGNP) with particle thickness in the range 4–30 nm, number of layers < 30, diameter of ~5–7 μm, aspect ratio ~ 240, specific surface area (SSA) from 30 to 40 m^2^/g, volume resistivity < 0.15 Ω·cm, and purity 90% were supplied by TimesNano, China. MWCNTs (NC7000) with outer diameter (OD) ~ 9.5 nm, length ~ 1.5 μm, purity 90%, aspect ratio 150, SSA = 250–300 m^2^/g, and volume resistivity 10–4 Ω·cm were picked up from Nanocyl, Sambreville, Belgium.

### 2.2. Output of Nanocomposite Test Films

All compositions were elaborated through direct mixing of polymer material and carbon nanopowder in the barrel of corotational twin screw extruder Thermo Scientific. The extrusion processing was carried out at 100 rpm screw velocity in ten-barrel temperature zones: 170, 175, 180, 180, 185, 185, 185, 180, 180, 180 °C. Afterwards, small quantities of pure PLA, monofiller and bifiller nanocomposite samples, each one in shape of pellets, were put up between two metal plates overlaid with Teflon sheets in order to avoid sticking of polymer material. Applying hot pressing at 180° there were obtained PLA and nanocomposite films with average thickness of ~400 μm.

### 2.3. Instrumental Methods

Quasi-static nanoindentation experiments, accelerated property mapping (XPM), nanoscratch, and dynamic mechanical testing (nanoDMA) have been performed on Hysitron TI 980 instrument (Bruker, Billerica, MA, USA) by using nanoDMA and 2D transducers. The 2D transducer assembly consists of normal and lateral forces and both transducers are equipped with Berkovich probe. A typical radius of camber for a standard Berkovich indenter is approximately 150 nm. The tip-area calibration was made using a standard specimen of fused quartz with known elastic modulus (69.6 GPa). Nanoindentation tests were made in load control mode by exerting peak force of 10,000 μN. The viscoelastic nature of the samples enforced an insertion of holding segment in the load function. Every single test consisted of 49 performed indents (7 × 7; spacing between indents 10 μm) in order to gather statistical data and to estimate the scattering of the load–displacement curves due to surface roughness. XPM analysis was done over sample surface area of 60 μm × 60 μm which is close to the utmost piezo scanner range. The procedure of accelerated property mapping was implemented by applying trapezoid load function with short segment loading, holding, and unloading time which were set to a few tenths of a second. Constant load scratch function with maximum force set to 1500 μN was applied during nanoscratch experiments. Dynamic nanomechanical analysis was implemented at frequency sweep mode of the nanoDMA function. In this mode, the oscillating dynamic force was superimposed on 1500 μN static force in the frequency range of 10–220 Hz. The average contact depth at that load depending on the tested composite sample was in the range of 400–500 nm. An extremely low displacement amplitude <0.1 nm during each one experiment ensured high accuracy and good repeatability of the results. The frequency sweep function has been applied by performing multiple cycles at every tread with a trend of lowering the number of cycles at the larger frequency values. Thus, an evaluation was made of the extent of energy dissipation within the nanocomposite structures or the damping behavior of the samples that indicates the accumulated damage on sample surface as an outcome of dynamic nanoindentation test. The viscoelastic nature of the analyzed specimens allowed an assignation of their time-dependent nanomechanical properties by measuring the phase lag δ between the applied load and the displacement response [12]. That lag is an important parameter outlining the stress relaxation behavior of the samples and providing a scope for nanodynamic mechanical analysis by receiving data about storage modulus, loss modulus, and tan δ.

The transmission electron microscopy (TEM) analysis was performed by using a FEI TECNAI G12 Spirit-Twin (LaB6 source) (Hillsboro, OR, USA) equipped with a FEI Eagle-4k charged coupling device camera, operating with an acceleration voltage of 120 kV. Prior to investigation, thin sections of the samples were cut at room temperature on a Leica EM UC6/FC6 ultramicrotome and placed on 400 mesh copper grids.

## 3. Results and Discussion

### 3.1. Quasi-Static Nanoindentation

**Figure 1:**
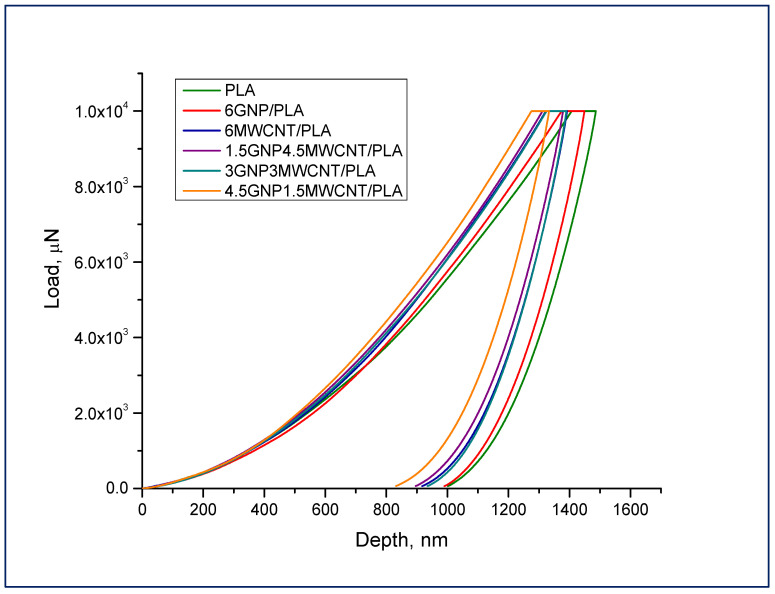
Nanoindentation curves of all composite specimens including pure PLA obtained at 10,000 μN peak force.

As a first experimental approach for surface characterization of nanocomposite films, the effective or reduced elastic modulus (*Er*) and the hardness (*H*) of the samples were determined by instrumented nanoscale indentation based on the Oliver–Pharr method [17]. The real time collection of depth and load data during quasi-static nanoindentation makes it a depth sensing technique. Reduced modulus of elasticity can be calculated by fitting the unloading portion of the load–displacement curve with power low function and finding the stiffness. Then, the mathematical relation between unloading stiffness and projected contact area under load aids to estimate *Er*. It has to be mentioned that the calculation of the reduced elastic modulus considers the fact of occurrence of elastic deformations both in the specimen and the indenter. The nanoindentation hardness is defined as the maximum load divided by the contact area of the indenter under peak load [18,19]. Average load–displacement curves representing various extent of penetration of the indenter within the composite samples can be seen in Figure 1. Applying 10 mN maximum force resulted in deeper displacement of above 1000 nm inside the material that allowed to gain more combined effect concerning elastic modulus and hardness. The lower maximum displacement at the curves corresponding to bifiller nanocomposite samples is an indication of higher resistance against the indenter movement inside the material and therefore it is a token for better nanomechanical properties of these hybrid composites primarily as regards to the non-modified PLA sample.

**Figure 2:**
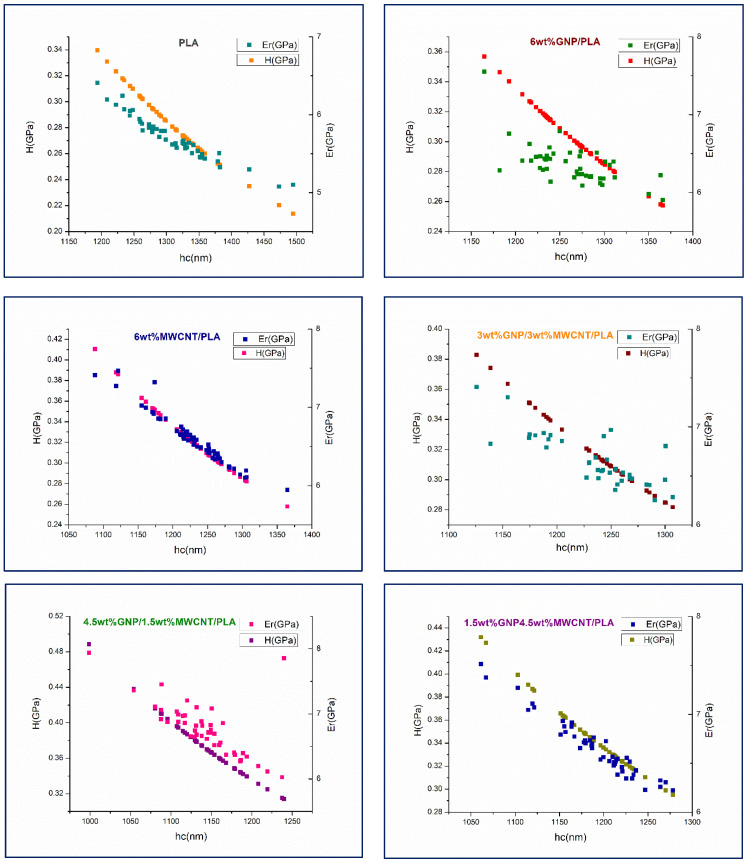
Nanoindentation hardness (*H*) and reduced elastic modulus (*Er*) of two monofiller and three bifiller composite specimens including neat PLA as a reference.

This finding is supported by the received nanoindentation data of all tested nanocomposites for the two main outputs—hardness and reduced elastic modulus, measured as a function of contact depth (hc), see Figure 2. Apparently, the bifiller nanocomposite films 1.5 wt%GNP4.5 wt%MWCNT/PLA and 4.5 wt%GNP1.5 wt%MWCNT/PLA show higher mechanical performance than the other samples, due to the occurrence of hybridization. The average values of *H* and *Er* for these compounds are 333 and 366 MPa, and 6.58 and 6.32 GPa, respectively. In comparison to the pure PLA, it was ascertained an improvement of about 22% regarding nanoindentation hardness and 14% with respect to the reduced elastic modulus in the values received for the bifiller composite samples. The reduction of the elastic modulus with increase of indentation depth at polymer materials is being explained by magnification of second order displacement gradients when using a Berkovich tip [20]. An attentive glance on the graphs in Figure 2 uncovers distinct indentation size effects and depth-dependent nanomechanical properties of the biopolymer composites concerning their elastic behavior revealed in the scatter of *Er* values. It seems that the reduced modulus of elasticity of the samples holding uppermost content of graphene nanoplatelets in the nanocomposite structure does not decrease so apparently with the increment of the contact depth. That lulled indentation size effect with respect to elastic deformation in the biopolymer composite die is most pronounced in the graphics of the compounds 6 wt%GNP/PLA and 3 wt%GNP3 wt%MWCNT/PLA, see Figure 2. As regards the hardness measurement, a constant nanomechanical behavior disclosing gradual decay of rigidity at higher penetration depth was logged for each one nanocomposite specimen. Having in mind that hardness, as a gaugeable parameter, is more sensitive to the top layer of a sample whence is coming the plastic behavior of the material, an inference can be made concerning the continuous relation of the composite plastic deformation to changes in the contact depth.

### 3.2. XPM Analysis

The core nature of the accelerated property mapping (XPM) method consists of high speed nanoindentation and mechanical property mapping of local surface area of a specimen in a really swift time spell [21]. In a sense, XPM takes what is normally an enforcement of single indent in expanding into a grid by using the equipped piezo scanner to rapidly swap position. Going through the procedure of XPM step by step, foremost the contact between the tip and the sample needs to be established by quick approach if tip to optic calibration has not been made in advance. The main benefit from nanoindentation of multiphase polymeric materials can be extracted by XPM technique enlarging over surface nanomechanical characteristics of different locations and getting contour map of the composite sample. Applying fast indentation can induce strain rate effect [22] and because of the fact that strain rate sensitivity is material specific, the best approach to deal with is to compare it against single quasi-static indent. Overview of the nanocomposites hardness and reduced elastic modulus data received from XPM testing indicates insignificant contrast with the values obtained by quasi-static nanoindentation that can be due also to the average roughness level of the composite films impeding to get high-consistent data.

**Figure 3:**
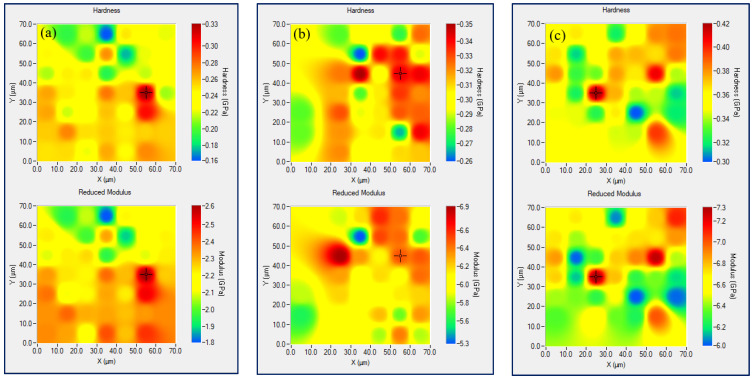

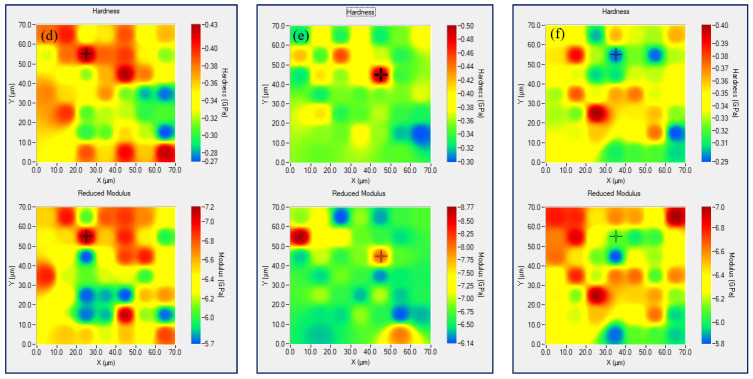
XPM plots of pure PLA (**a**), 6 wt%GNP/PLA (**b**), 6 wt%MWCNT/PLA (**c**), 4.5 wt%GNP1.5 wt%MWCNT/PLA (**d**), 3 wt%GNP3 wt%MWCNT/PLA (**e**) and 1.5 wt%GNP4.5 wt%MWCNT/PLA (**f**).

This assumption was supported later on by performing in-situ SPM imaging of samples’ nanoscratch surface area. Drawing of the superficial mechanical features of nanocomposite sample represents actually an evaluation of the surface homogeneity of that material as regards the nanomechanical behavior of precisely marked composite surface area subjected to XPM testing. Experimental property mapping plots, performed in load control indentation mode and showing the uniformity of surface hardness and elasticity of nanocomposite films including pure PLA, are presented in Figure 3.

**Figure 4:**
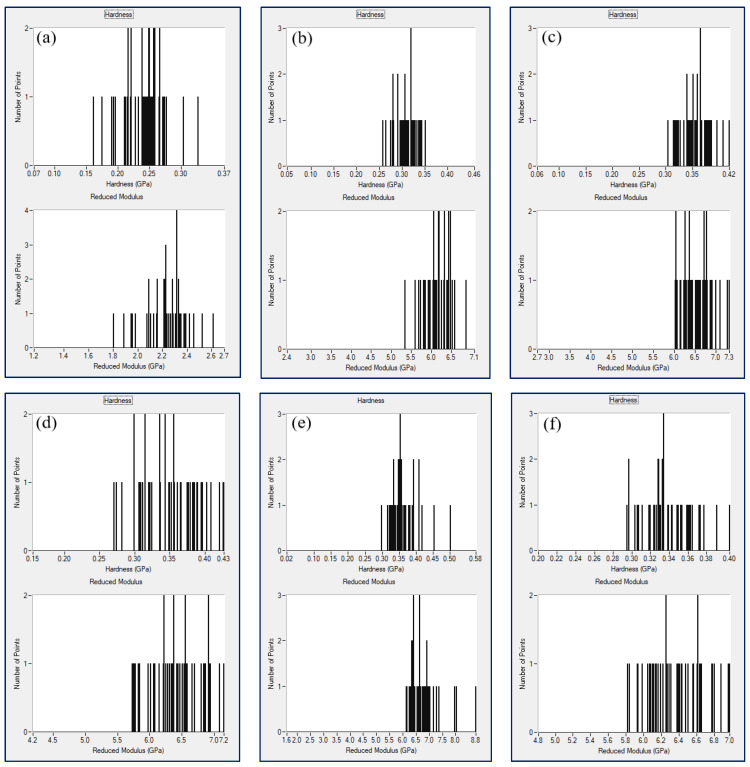
XPM histogram graphics of pure PLA (**a**), 6 wt%GNP/PLA (**b**), 6 wt%MWCNT/PLA (**c**), 4.5 wt%GNP1.5 wt%MWCNT/PLA (**d**), 3 wt%GNP3 wt%MWCNT/PLA (**e**) and 1.5 wt%GNP4.5 wt%MWCNT/PLA (**f**).

Regardless of the level of hardness and elastic modulus, registered at the colorful bar right positioned in every property map, the alignment of the surface mechanical features looks similar for all samples except the bifiller composite 3 wt%GNP3 wt%MWCNT/PLA that apparently possesses most homogeneous surface properties compared to the other XPM plots, see Figure 3e. Thus, one can conclude about the presence of synergistic effect as an outcome of the hybrid composite structure formed by two carbon nanofillers and the polymer chains. This is valid not only regarding the values of hardness and elastic modulus but as well with respect to the mechanical surface property mapping of the bifiller nanocomposite. Histogram distribution of the nanomechanical parameters corresponding to each one XPM plot can be seen in Figure 4. Narrow dispersion of the lines corresponding to hardness and reduced elastic modulus can be noticed primarily for the sample 3 wt%GNP3 wt%MWCNT/PLA. This is in good compliance, as expected, with the nanomechanical property mapping data presented in Figure 3e. Another nanocomposite specimen showing apparent homogeneous surface mechanical behavior, especially with respect to the values of hardness, was found to be the sample owing 6 wt%GNP in the PLA matrix, see Figure 4b. Though the nanomechanical parameters of the composite 6wt%GNP/PLA are relatively lower compared to the bifiller samples, XPM plot and histogram analysis still indicate good dispersion of graphene nanoplatelets in the polymer structure. That fact approves the reliability of the applied preparation method having in mind the tendency of GNPs to agglomerate [23] that makes the receiving of balanced nanocomposite material not easy to achieve.

### 3.3. Nanoscratch Testing

**Figure 5:**
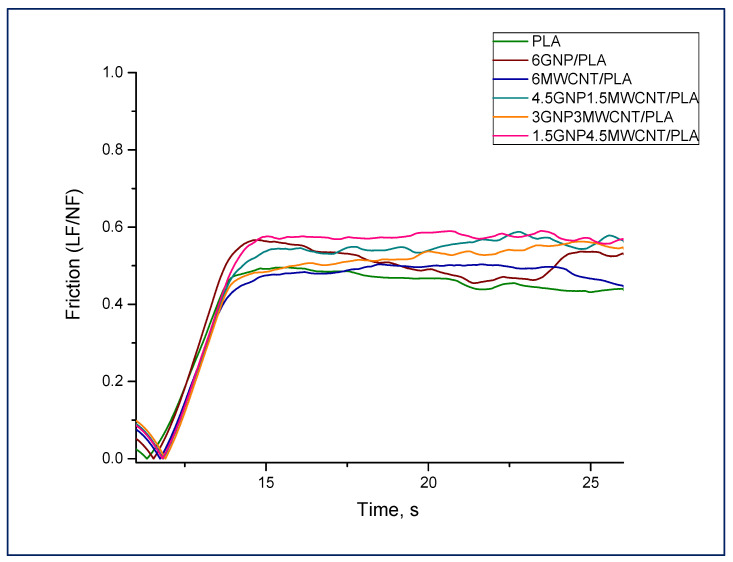
Friction behavior at nanoscratch testing of monofiller and bifiller nanocomposites including pure PLA as a reference.

As a nanoscratch experiment is one of the essential tools for assessing the nanomechanical and tribological properties of composite materials, it was enclosed as a part of the specimens’ characterization. The test procedure of the selected constant load scratch function is based on the acquisition of several main parameters as a result of an accurate experimental completion providing reliable data [24]. In order to define nanocomposite surface hardness, the friction behavior of the samples during performance of nanoscratch is presented by using the precise segment from the instrumental load function that reflects the sheer motion of the indenter over the composite surface (Figure 5). In fact, the average values of the steady state section of the curves, where the regime of constant scratch is plotted, are indicative for obtaining of coefficient of friction (COF = LF/NF). The rate of the ascertained lateral force depends on the scratch resistance pursued by the material against the movement of the pyramidal Berkovich probe along the sample surface. Therefore, the coefficient of friction can be used to evaluate the nanocomposite hardness by measuring the surface resistance versus nanoscratch, as mentioned in our recent article [24].

It should be noticed that the bifiller films demonstrated higher ability to endure strain caused by the indenter scratching move than the monofiller samples, see Figure 5. Hence, these hybrid structures own higher nanomechanical hardness as already confirmed by quasi-static nanoindentation.

**Figure 6:**
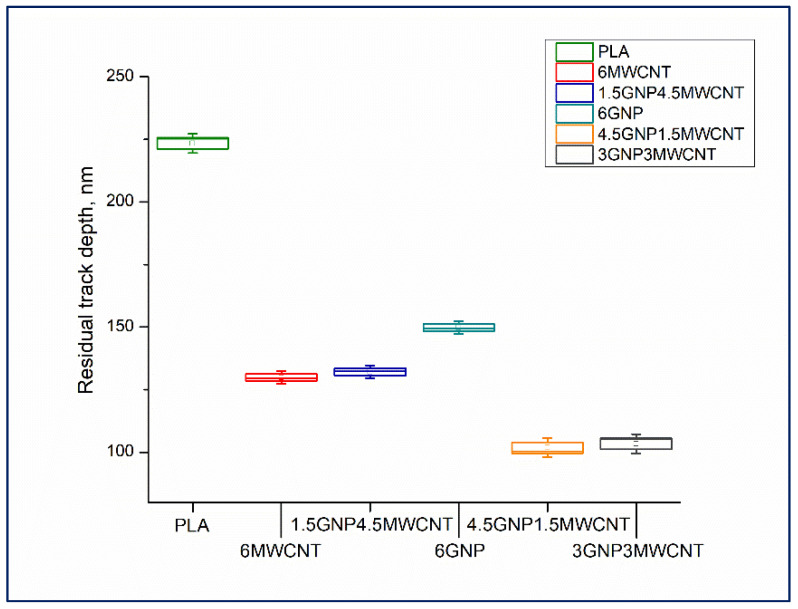
Remnant track depths of nanoscratches performed over monofiller and bifiller composite samples including pure PLA as reference.

Retrieve of scratch hardness should mean shallower depth of the notch left by the indenter on the nanocomposite surface. The samples’ nanomechanical behavior was estimated by in-situ SPM imaging of the performed nanoscratch test and following topography investigation of the trace. Capturing high resolution SPM images of the experimental surface areas allowed to measure the residual track depths of the scratched specimens, see the box chart in Figure 6.

**Figure 7:**
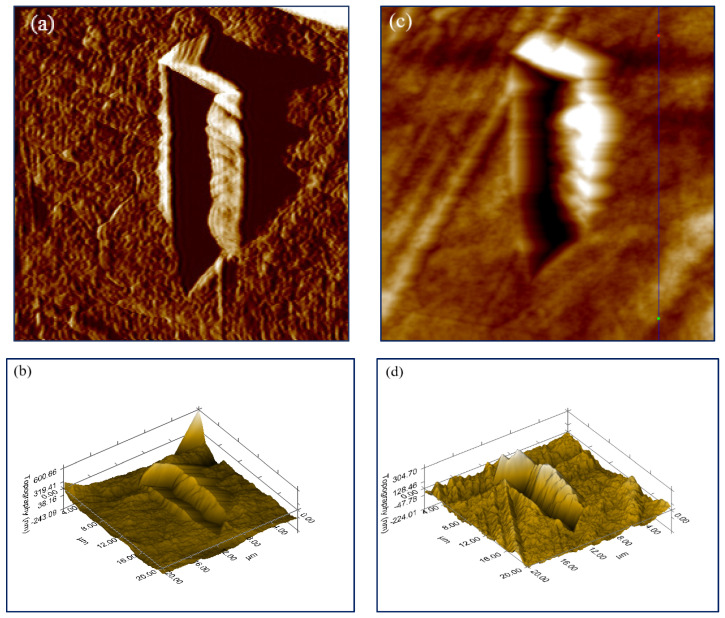
In-situ SPM images of gradient forward (**a**) and 3D (**b**) scans over 20 μm × 20 μm surface area of nanoscratched 3 wt%GNP3 wt%MWCNT/PLA composite sample, and topography forward (**c**) and 3D (**d**) scans over 20 μm × 20 μm surface area of nanoscratched 6 wt%GNP/PLA composite sample.

The scope of the plotted data for each one nanocomposite thin film is based on the examination of three scratch tests for statistical purposes. As expected, the bifiller composite samples along with the monofiller compound having carbon content of 6 wt% MWCNT were found to possess lower residual groove depth contrasted to the samples 6wt%GNP/PLA and neat PLA. This is in good agreement with the results established for scratch resistance and it seems that these nanomechanical features are closely related. An illustration of outstanding gradient and topography forward images as well as 3D morphology pictures acquired through applying of in-situ SPM scanning mode can be seen in Figure 7. The presented 3D images disclose unarguably deeper groove belonging to the monofiller composite surface (Figure 7d) and shallower indenter trace formed on the thin film surface of the bifiller composite sample (Figure 7b), both created by nanoscratch test made with the same constant load function designed by setting up a peak force value of 1500 μN. Considering the significance of the surface average roughness, calculated by processing of an in-situ SPM topography forward image (Figure 7c), it was found that the bifiller nanocomposites have higher surface flatness than the monofiller samples including the neat PLA, see Table 1. This is probably due to the synergistic effect of GNPs and MWCNTs on the nanocomposite structure modifying the interconnected hybrid framework at the interface beneath the upper biopolymer thin layer [25]. It has to be mentioned that the hot pressing technique of thin films’ preparation apparently provides samples with higher average roughness than the 3D printing method [24] but in the current nanomechanical investigation the impact of the carbon nanofillers on the composite surface properties is ascertained to be more considerable. The asperities in the plain of the 3D plot of the specimen 6 wt%GNP/PLA (Figure 7d) are notably greater contrasted to the top side of the bifiller composite 3 wt%GNP3 wt%MWCNT/PLA (Figure 7b). As well-known, there is a critical correlation between the reliability of the nanoscratch experiments and the smooth and clean composite surface [26]. The detected lateral force depends on the Van der Waals forces experienced by the nanoindenter during the contact with the sample which is linked to the local tilt of the surface bumps [27]. Therefore, the reduced average roughness of the bifiller specimens, being in the range of 14–18 nm (Table 1), allows a flatter conduct of the nanoscratch testing.

Another essential feature concerning the mechanism depicting the nanoscratch behavior of composite materials is the height of pile-up on the rims of the groove left by the indenter tip, see Figure 7b,d. It is obvious that the higher amount of material pile-up along the track on the surface of the monofiller sample possessing 6 wt%GNP in the polymer matrix is in full accordance with the deeper notch brought by the probe. This finding confirms the lower resistance against scratch of that sample compared to the bifiller nanocomposite where the quantity of plastically torn material accumulated on the groove sides and pushed ahead by the plowing indenter is less significant (Figure 7b).

### 3.4. Nanodynamic Mechanical Analysis

Dynamic nanoindentation testing was made in order to evaluate the viscoelastic properties of the nanostructured polymer-based composite films. The application of frequency sweep dynamic load function in nanoDMA actuating mode of a standard transducer aided in the complete understanding of the relative influence of changing dynamic frequency as test parameter during nanoindentation experiment. That variation in harmonic frequency from 10 to 220 Hz with keeping constant load amplitude of 1 μN was performed to examine its impact on the estimated elastic stiffness parameters. The storage modulus (*E*′) determines the nanocomposite strain rate sensitivity and can be designated as a characteristic of the material’s elastic behavior. Actually *E*′ is the real part of the complex modulus (E*), the latter being function of the phase shift δ, and is described as a capacity of the composite sample to heap potential energy that is subsequently detached in the strain process, see Equation (1). The loss modulus (*E*′′) represents the imaginary part of E* and this is the viscoelastic parameter responsible for internal damping within the composite matrix [28] reflecting the amount of instantly dissipated energy, see Equation (2).

(1) $$E^{\prime }=\frac{\sigma }{\varepsilon }\left(\cos\;\delta \right)$$

(2) $$E^{\prime \prime }=\frac{\sigma }{\varepsilon }\left(\sin\;\delta \right)$$

The ratio between the viscoelastic component *E*′′ and the elastic component *E*′ forms the loss factor (tan δ) which is commonly used to measure the oscillatory strain response of the material caused by the exerted dynamic mechanical force during nanoindentation.

**Figure 8:**
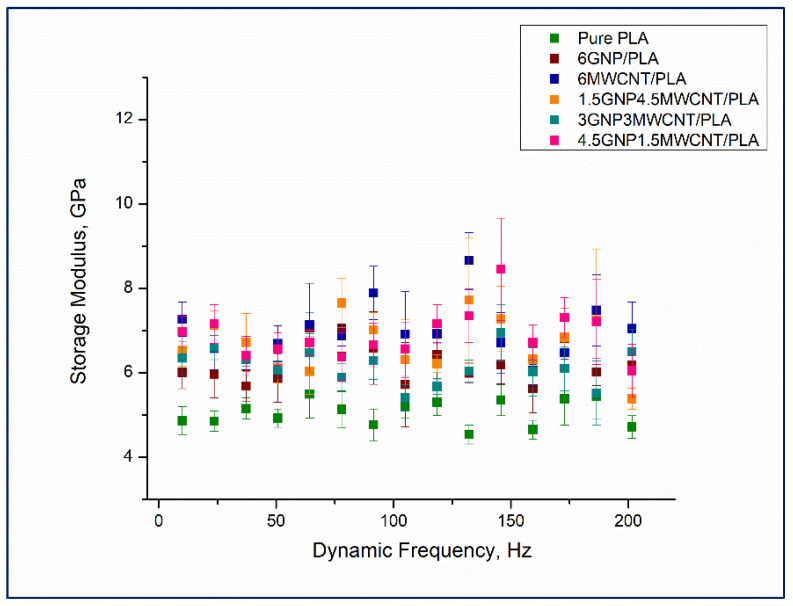
Storage modulus versus dynamic frequency for the whole series of nanocomposite samples including neat PLA.

**Figure 9:**
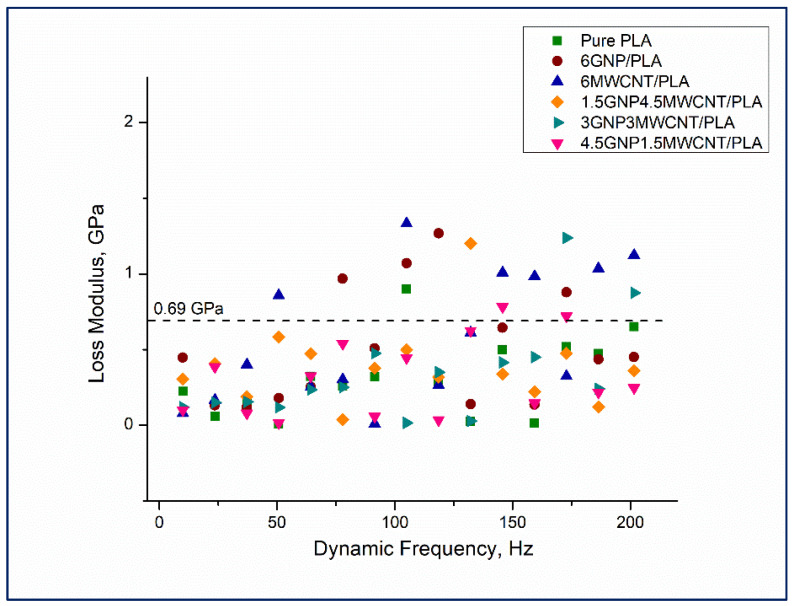
Loss modulus versus dynamic frequency for the whole series of nanocomposite samples including neat PLA.

**Figure 10:**
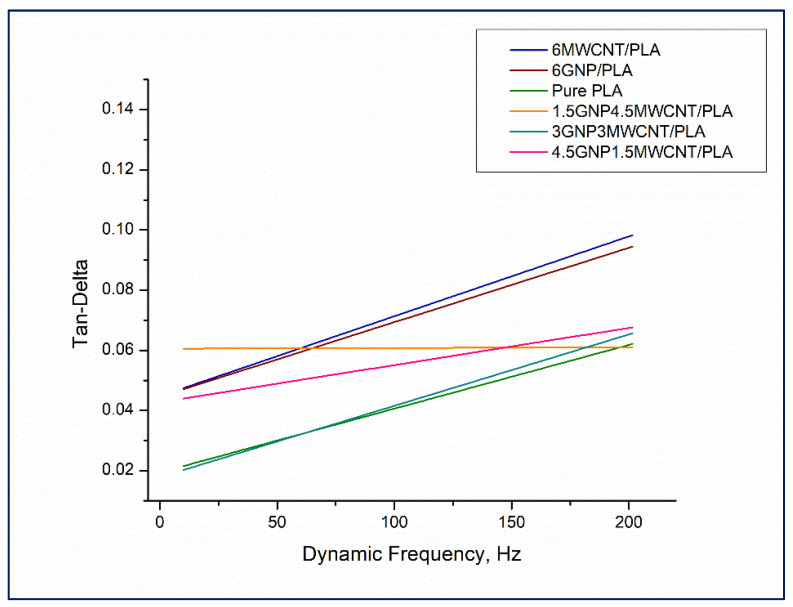
Tan δ versus dynamic frequency for the whole series of nanocomposite samples including neat PLA.

Results from nanoDMA testing of monofiller and bifiller nanocomposite thin films in terms of storage modulus, loss modulus, and tan δ as a function of the dynamic frequency are shown in Figure 8, Figure 9 and Figure 10. Observing the values of the storage modulus, depicted in Figure 8, it can be noticed that some contrasts in elastic stiffness between the sample compositions are evident. All nanocomposites have higher storage modulus in comparison to the pristine polymer and the good elastic response of the specimens 1.5 wt%GNP4.5 wt%MWCNT/PLA, 4.5 wt%GNP1.5 wt%MWCNT/PLA, and 6 wt%MWCNT/PLA under cyclic loading, see the square colorful symbols in Figure 8, is in full agreement with the received quasi-static nanoindentation data which was discussed earlier in the paper. It seems that the reinforcement of PLA with carbon nanoparticles redounds toward an increment of the storage modulus whereof it follows that the elastic behavior of the nanocomposites is probably influenced by interactions at the interface carbon nanofiller–polymer chain. Bimodal presentation of loss modulus of the samples can be seen in Figure 9.

The plotted values are virtually separated in two distinct graph zones, see the dash line. The region under loss moduli of 0.69 GPa has as a characteristic a pile up of overlapping viscoelastic property data regardless of dynamic frequency and carbon nanofillers loading in the composite structure. The upper plot area unifying loss moduli values in the range 0.69–1.34 GPa could be explained in a different manner. Most of the loss modulus data points falling in that graph section belong to the monofiller nanocomposites 6wt%MWCNT/PLA and 6wt%GNP/PLA. This mechanical behavior is observed in the frequency range above 50 Hz and can be due to higher internal damping in these samples as a lineal outcome of good carbon nanoparticle dispersion in the composite matrix leading to an increase of the dissipated energy during deformation. The relative lack of stacked graphene nanoplatelets and entangled carbon nanotubes makes the composite material more sustainable to continuous cyclic loading by hampering crack formation via substantial reduction of damage mechanisms [29]. In terms of more comprehensible drawing in the trend of tan δ values as a function of dynamic frequency, a linear curve fitting of the loss factor data obtained for each one nanocomposite thin film has been made (Figure 10). Except the fit line of the data points of the sample 1.5 wt%GNP4.5 wt%MWCNT/PLA, all other tan δ curves rise with the increment of the harmonic frequency. This distinct damping behavior of that specific bifiller specimen can be assigned to a higher dynamic stiffness of the hybrid material throughout the continuous loading process by varying the dynamic frequencies. In fact, the nanocomposite 1.5 wt%GNP4.5 wt%MWCNT/PLA demonstrated good ability to resist elastic deformation at quasi-static nanoindentation that correlates with the performed nanoscale dynamic mechanical analysis. Constant tan δ values could signify standing viscoelastic properties of the material and high endurance against damage in the composite matrix due to the total number of 1330 cycles laid down in the frequency sweep dynamic load function. Interestingly, the tan δ curves of the monofiller nanocomposite samples are positioned above the curves of the bifiller specimens, see Figure 10. A probable interpretation can be related to the presence of uniform dispersion of the carbon nanofiller in the PLA die enhancing the ductile properties of these composites and therefore leading to a greater level of energy dissipation as a result of the experienced plastic strain.

Overall, the three bifiller nanocomposites have lower tan δ values compared to the monofiller samples and further exhibiting an individual viscoelastic behavior with variation of tan δ in the range 0.02–0.06. The rising slope of the curves glancing the trend of tan δ result data received for the composites 4.5 wt%GNP1.5 wt%MWCNT/PLA and 3 wt%GNP3 wt%MWCNT/PLA is an indication of a dependence of the plastic properties of these bifiller samples on the higher loading rate in a frequency sweep test. It has to be mentioned that the ratio between the percentage content of GNPs and MWCNTs in the polymer matrix has an impact on the ratio of viscoelastic to elastic response in the material in the course of shifting the dynamic frequency of applied oscillating force to higher Hertz units.

### 3.5. TEM Analysis

**Figure 11:**
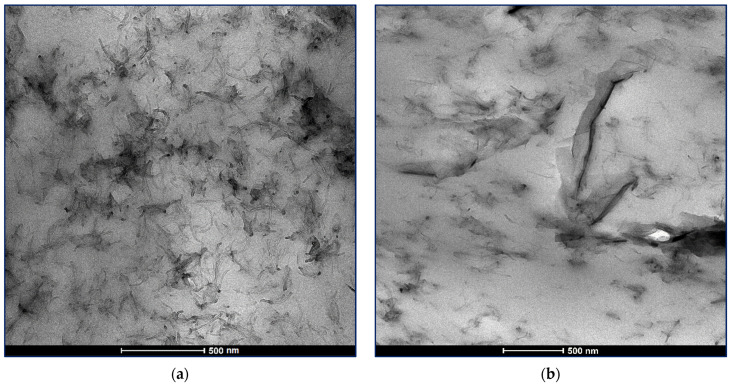
TEM micrographs of the bifiller nanocomposite films 1.5 wt%GNP4.5 wt%MWCNT/PLA (**a**) and 4.5 wt%GNP1.5 wt%MWCNT/PLA (**b**).

The good dispersion of the carbon nanofillers in a polymer matrix is crucial for the receiving of nanocomposites with reliable nanomechanical characteristics [30]. High resolution TEM images of the PLA-based bifiller nanocomposite films having accordingly 1.5 wt%GNP4.5 wt%MWCNT and 4.5 wt%GNP1.5 wt%MWCNT carbon nanofiller content are presented in Figure 11. Study of the TEM micrographs discloses a fine correlation between the high level of carbon nanoparticles distribution in the PLA die and the formation of sheer hybrid nanocomposite structure leading to emergence of synergism and improvement in the nanomechanical behavior of the composite samples, as shown in the nanoindentation and nanoscratch analyses. It can be seen that the carbon nanotubes and the graphene nanoplatelets build up an interconnected network in the polymer matrix favoring the advanced application of the novel nanocomposite materials.

## 4. Conclusions

Summarizing the wide range of overwhelming nanomechanical data obtained over the attentively designed and elaborated biodegradable nanocomposite films, it should be noted the high performance regarding their elastic properties and scratch resistance behavior. In general, quasi-static nanoindentation, nanoscratch and XPM testing revealed some tints of synergy between GNPs and MWCNTs in the hybrid nanocomposite structure lending impact on the exceptional nanomechanical performance of the bifiller samples. In terms of nanodynamic mechanical investigation of the composites the accent fell over the measuring of elastic stiffness and loss factor parameter whereof it was made an inference for a solid viscoelastic behavior of the specimens subjected to extended cyclic loading. A thoughtful reading at the nanoscale surface characterization of PLA-based nanocomposites suggests that the mechanism of mechanical reinforcement is probably influenced by the anisotropic shape, the specific surface area, and the homogeneous dispersion of the incorporated carbon nanofillers. This assumption allows to tune several diverse and at the same time key nanomechanical features as indentation size effect, residual scratch depth and internal damping (tan δ) by selective loading of GNPs and/or MWCNTs content in the polymer matrix.

## Figures and Tables

**Table 1:** Average surface roughness received by in-situ SPM imaging of nanocomposite surface area subjected to nanoscratch testing.

No	Nanocomposite Surface	Average Roughness, nm
1	PLA	36.1
2	6 wt%GNP/PLA	25.0
3	6 wt%MWCNT/PLA	24.0
4	4.5 wt%GNP1.5 wt%MWCNT/PLA	15.1
5	3 wt%GNP3 wt%MWCNT/PLA	18.0
6	1.5 wt%GNP4.5 wt%MWCNT/PLA	14.3

## Data Availability

All raw data that are in the fundamental of the results presented in this study can be provided by the corresponding author on request.
